# Application of information from external data to correct for collider bias in a Covid-19 hospitalised cohort

**DOI:** 10.1186/s12874-023-02129-7

**Published:** 2024-07-16

**Authors:** Annastazia Learoyd, Jennifer Nicholas, Nicholas Hart, Abdel Douiri

**Affiliations:** 1 School of Life Course and Population Sciences, King College London, London, UK; 2 Department of Medical Statistics, London School of Hygiene and Tropical Medicine, London, UK; 3 Lane Fox Clinical Respiratory Physiology Research Centre, Guy’s & St Thomas’ NHS Foundation Trust, London, UK; 4 Centre for Human and Applied Physiological Sciences, King’s College London, London, UK

**Keywords:** Collider bias, Covid-19, Cohort, Inverse probability weighting

## Abstract

**Background:**

Throughout the Covid-19 pandemic, researchers have made use of electronic health records to research this disease in a rapidly evolving environment of questions and discoveries. These studies are prone to collider bias as they restrict the population of Covid-19 patients to only those with severe disease. Inverse probability weighting is typically used to correct for this bias but requires information from the unrestricted population. Using electronic health records from a South London NHS trust, this work demonstrates a method to correct for collider bias using externally sourced data while examining the relationship between minority ethnicities and poor Covid-19 outcomes.

**Methods:**

The probability of inclusion within the observed hospitalised cohort was modelled based on estimates from published national data. The model described the relationship between patient ethnicity, hospitalisation, and death due to Covid-19 – a relationship suggested to be susceptible to collider bias. The obtained probabilities (as applied to the observed patient cohort) were used as inverse probability weights in survival analysis examining ethnicity (and covariates) as a risk factor for death due to Covid-19.

**Results:**

Within the observed cohort, unweighted analysis of survival suggested a reduced risk of death in those of Black ethnicity – differing from the published literature. Applying inverse probability weights to this analysis amended this aberrant result to one more compatible with the literature. This effect was consistent when the analysis was applied to patients within only the first wave of Covid-19 and across two waves of Covid-19 and was robust against adjustments to the modelled relationship between hospitalisation, patient ethnicity, and death due to Covid-19 made as part of a sensitivity analysis.

**Conclusions:**

In conclusion, this analysis demonstrates the feasibility of using external publications to correct for collider bias (or other forms of selection bias) induced by the restriction of a population to a hospitalised cohort using an example from the recent Covid-19 pandemic.

**Supplementary Information:**

The online version contains supplementary material available at 10.1186/s12874-023-02129-7.

## Background

 With the start of the Covid-19 pandemic came an initial rush to research and publish findings about this novel disease. But a year into the pandemic reflective publications started to highlight the high risk of bias in these early studies [1–4]. Many Covid-19 studies are observational studies utilising electronic health records and/or hospitalised cohorts of patients with the aim of investigating the average causal effect of various demographic and clinical variables on Covid-19 outcomes such as death. These avenues for patient recruitment/data collection are prone to selection bias - bias that has been demonstrated to induce a relationship between allergy medications and increased Covid-19 testing [5] and suggested to induce relationships between smoking and a reduced risk of Covid-19 infection [6], or between ACE inhibitor use and Covid-19 severity [2].

Collider bias, as a form of selection bias, has garnered much attention [2]. This is induced when both the exposure and outcome of interest impact a third variable which is conditioned upon – such as a variable determining sample selection. Within the common observational studies of hospitalised cohorts [7–14] these associations are likely. Covid-19 severity (typically measured by ICU admission or mortality) is associated with hospitalisation – only the ~ 15% most severe cases will require hospitalisation and advanced medical care [15]. Therefore, one of the two required associations for collider bias (outcome to third variable: hospitalisation) is implicitly met in many Covid-19 studies. Only an association between the exposure and hospitalisation is required to create biased results.

Studies of hospitalised patients could provide valuable insight into novel diseases such as Covid-19 as long as bias can be minimised. Classic corrections for collider bias involve using inverse probability weighting (IPW) based on the probability of inclusion into the study [2, 16]. IPW allows the characteristics of the sample to be adjusted to become more representative of the target population, breaking the conditioning on the sample selection variable. However, these methods can only be easily applied to nested studies where key variables are only examined in a subset of individuals and the probability of selection into this subset is known or can be accurately modelled based on measured covariates. As Covid-19 studies are typically opportunistic, non-nested studies, acquiring such knowledge is difficult – but maybe not impossible. A wealth of electronic health data is available related to Covid-19. In the UK this includes larger national cohorts established prior to the Covid-19 pandemic that continued to collect and analyse health records during this time [17–20] as accessible data from the NHS and UK government on numbers of Covid-19 cases, hospitalisations, and death by NHS trusts/local regions [21–24]. While these data does not provide an exact replacement for the missing information on the probability of Covid-19 hospitalisation from the local community, it could be used to estimate likely probabilities.

Thompson and Arah [25] have described a method of applying external data to model the probability of inclusion in a study where data from unobserved individuals is unknown. This method models the probability of inclusion based on the exposure and the outcome using an external dataset (and allows for additional adjustment by relevant covariates). Simulations showed that IPW developed using this model could accurately obtain the ‘true’ adjusted odds ratios for a dataset and were shown to be robust against misspecification of model parameters. We propose that the wealth of national data on Covid-19 measures allows the application of this method to data from hospitalised cohorts, allowing correction for collider bias in these susceptible studies.

To this end, this article demonstrates the use of UK national data on Covid-19 cases and deaths to model the probability of hospitalisation which can be applied as IPW to a regional hospitalised cohort to correct for selection biases including collider bias. We also demonstrate that the obtained results are mostly robust to misspecification in the developed model – allowing for differences in the probability of Covid-19 associated hospitalisation between national and local populations which may be unaccounted for.

### A relationship with likely collider bias

**Fig. 1:**
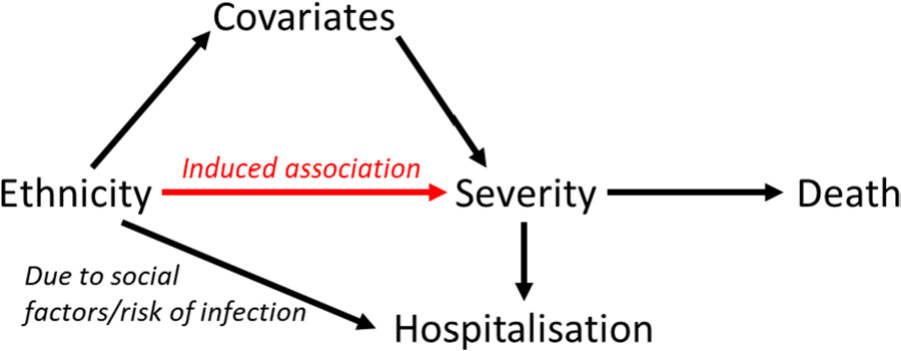
Brief Directed Acyclic Graph demonstrating the possible relationship between ethnicity exposure) and death (outcome) with the presence of the collider hospitalisation. This figure is a condensed version of the Directed Acyclic Graphs provided in Learoyd et al. [29] and in Appendix Fig. 1

Demonstrating the application of Thompson and Arah’s method requires the selection of an exposure and outcome to explore. Minority ethnicities during the early pandemic were described as being at increased risk of death due to Covid-19 [11, 17, 20, 26, 27]. Cultural factors, occupation preferences, and social inequalities such as poor housing and reduced access to healthcare mean that these ethnic groups are more likely to be infected with Covid-19 and as a result are more likely to be hospitalised with Covid-19 [11, 19, 20, 28]. Ethnicity therefore provides an ideal exposure that is likely to induce some form of collider bias when examined within a hospitalised cohort (Fig. 1). An outcome of mortality during hospitalisation will be used – matching the outcome commonly seen in Covid-19 studies.

## Methods

### Patient population

To explore the correction for collider bias utilising external data, data from Covid-19 positive patients admitted to Guy’s and St. Thomas’ NHS Foundation Trust (GSTT) between 20th February 2020 and 24th May 2021 was used. Patients were restricted to adults (18+) admitted from the community after 28th January 2020 (the date of the first known Covid-19 case in the UK) with a positive Covid-19 test within 28 days of admission who had known age, sex, Index of Multiple Deprivation (IMD). IMD is a relative measure of deprivation for small regional areas in the UK based on 7 domains of deprivation identified by the patient’s current address [30]. Medical history for these patients was collated from 6 linked databased and categorised as presence/absence of cardiovascular disease (stroke, transient ischaemic attack, atrial fibrillation, congestive heart failure, ischaemic heart disease, valve disease, peripheral artery disease or atherosclerotic disease), diabetes mellitus, chronic kidney disease, chronic liver disease, and chronic obstructive pulmonary disease/emphysema. The application of “do not attempt resuscitation” (DNR) orders was also extracted along with the date of application. These covariates were chosen for inclusion in this analysis based on known association with Covid-19 outcomes and known links to ethnicity. Ethnicity was categorized as White (British, European, Other), Black (African, Caribbean), Asian (South, South-East, and East Asian), Mixed/Other (Middle Eastern, South American, and Mixed) and Unknown (or not reported). The cohort characteristics are shown in the appendix (Appendix Page 8, Appendix Table 1 & Appendix Fig. 2).

Patients were categorised into two Covid-19 waves based on admission date for analysis purposes. Admission was designated as belonging to the first (until 31st August 2020) or second (from 1st September 2020) wave of Covid-19 matching timepoints used to analyse the OPENSAFELY cohort. This categorisation, along with ethnicity and survival status, informed the IPW (described below) given to each patient.

### Statistical modelling

Time to death was analysed using Cox proportional hazards models with discharge as a competing event and robust standard errors. The association of ethnicity with death was assessed using three model iterations: (1) unadjusted (with no additional covariates), (2) adjusted for all described covariates, (3) adjusted for covariates and using IPW based on the probabilities derived below.

#### Part 1: Estimating the probability of Covid-19 associated hospitalisation in the first wave from national data

Estimating the necessary probabilities for IPW (following the principles laid out by Thompson and Arah [25]) requires an estimation of the risk of hospitalisation overall and estimation of the risk ratios (RRs) describing the relationship between hospitalisation and different ethnic groups stratified by survival status. These can be derived from a generalised linear model (GLM) for the probability of hospitalisation (Eq. 1).


1


Importantly the relationship between ethnicity and Covid-19 outcomes – including hospitalisation and death – has been reported for a national cohort in the UK. Mathur et al. [20] completed an analysis of the OPENSAFELY platform which holds electronic health record data from individuals registered with English primary care practices allowing an ideal reference point for estimating the probability of hospitalisation in different ethnic groups. Summary-level data from this publication included the number of adults from 16 different ethnic categories who were hospitalised with Covid-19 or experienced death associated with Covid-19 across the first (1st February to 3rd August 2020) and partial-second (1st September to 31st December 2020) wave of Covid-19.

Cross-tabulation of hospitalisation and survival status was not provided by Mathur et al. and so it was assumed initially that 90% of individuals who died were hospitalised prior to death (regardless of ethnicity). This assumption is based on the understanding that those who died had more severe disease and were more likely to be hospitalised along with an expectation not all deaths would occur in hospital. Based on this assumption and the summarised data estimates for the parameters in Eq. 1 could be obtained (Table 1) allowing the probability of hospitalisation with Covid-19 to be modelled for each ethnic group stratified by survival status. The inverse of these probabilities formed the sampling weights used as IPW allowing the conditional causal effect effect of ethnicity to be estimated as applied to a pseudo-population more representative of that in the community.

**Table 1:** The risk of hospitalisation with Covid-19 for each ethnic group in the first wave of Covid-19 as obtained from published analysis of the OPENSAFELY platform [20] and fitted parameters for the model in Eq. 1 that can be estimated from the OPENSAFELY data. OIM std error = standard errors derived from the observed information matrix variance estimators

Ethnicity	Hypothesised risk of hospitalisation		Exponentiated fitted parameters ± OIM std. err.			
	Survived	Died				
White	0.00126	Assumed 0.90	${\widehat{\gamma }}_{0}$	0.0012 ± 0.0000	${\widehat{\gamma }}_{1}$	823.70 ± 7.265
Black	0.00238	Assumed 0.90	${\widehat{\lambda }}_{a1}$	1.9190 ± 0.0704	${\widehat{\lambda }}_{b1}$	0.5211 ± 0.0194
Asian	0.00197	Assumed 0.90	${\widehat{\lambda }}_{a2}$	1.5922 ± 0.0370	${\widehat{\lambda }}_{b2}$	0.6280 ± 0.0148
Mixed/Other	0.00155	Assumed 0.90	${\widehat{\lambda }}_{a3}$	1.2581 ± 0.0531	${\widehat{\lambda }}_{b3}$	0.7949 ± 0.0342
Unknown	0.00101	Assumed 0.90	${\widehat{\lambda }}_{a4}$	0.7944 ± 0.0139	${\widehat{\lambda }}_{b4}$	1.2587 ± 0.0222

#### Part 2: Sensitivity analysis examining the effect of changes in estimated probabilities to match the local population

As the IPW used in this analysis is externally obtained data, it is important to validate it’s use by examining whether misspecification of the model parameters describing the association between ethnicity/death and hospitalisation would alter the obtained results.

Additional sources of data describing the risk of hospitalisation for specific ethnicities and survival status can be obtained from a combination of NHS and Office of National Statistics (ONS) sources [21–24]. Examination of these data may identify the extent to which the probability of hospitalisation as calculated for the national cohort examined by OPENSAFELY [20] differed for a local London-based (GSTT) cohort and so the likely degree of misspecification that is present for the model parameters in Eq. 1. Two comparisons were made: one utilizing data specific to the GSTT catchment area (i.e. NHS reported cases for GSTT; ONS estimates for Lambeth, Lewisham and Southwark) (Table 2) and one using data relevant to the Greater London area (Appendix Table 2).

**Table 2:** Risk of Covid-19 associated hospitalisation for those with different ethnicities and survival status as calculated for the OPENSAFELY cohort [20] and the GSTT catchment area. Data informing the risk for the GSTT catchment area indicated in italics. Risk Ratios (RR) compare risks to applicable reference groups: No Death or White ethnicity. Differences in ln(Risk) or ln(RR) informed the level of misspecification applied to each indicated parameter (Informs column) during sensitivity analysis

Variable/Interaction	OPENSAFELY		GSTT		Difference in ln(RR)	Informs
	Risk	RR	Risk	RR		
		*(NHS reported cases/ONS estimated population)*				
Overall in Wave 1	0.00187		0.00177		**ln(Risk)=-0.05**	${\widehat{\gamma }}_{0}$
Survival status (Outcome)	No Death: *(NHS reported discharges/ONS estimated population)* Death: *(ONS death occurrences involving COVID-19)*					
No Death	0.00121		0.00104			
Death	Assumed 0.90	744	0.76359	734	**-0.01**	${\widehat{\gamma }}_{1}$
Ethnicity (Exposure)		*(Analysed cohort/ONS estimated population)*				
White	0.00185		0.00047			
Black	0.00307	1.66	0.00118	2.48	**0.40**	${\widehat{\lambda }}_{a1}$
Asian	0.00257	1.39	0.00089	1.87	**0.30**	${\widehat{\lambda }}_{a2}$
Mixed/Other	0.00188	1.02	0.00054	1.14	**0.11**	${\widehat{\lambda }}_{a3}$

These comparisons found comparable risks of hospitalisation overall in the OPENSAFELY cohort compared to that expected for London (informing misspecification of ${\widehat{\gamma }}_{0}$). the assumption that 90% of patients who died from Covid-19 were hospitalised (informing ${\widehat{\gamma }}_{1}$, RR comparing those who died vs., those who survived) was shown to be incorrect. ONS provided data on the place of Covid-19 associated death estimated that only 72–82% of individuals in London died in hospital.

Rates of hospitalisation were not stratified by ethnicity. To allow the degree of misspecification to be explored for ${\widehat{\varvec{\lambda }}}_{\varvec{a}}$ (RRs comparing ethnic groups in those who didn’t die) to be determined, the risk of hospitalisation for each ethnicity was estimated from the GSTT cohort residing in the catchment area divided by the ONS population estimates for the same area. The relative risk of hospitalisation in minority ethnicities compared to White ethnicity was larger for the GSTT cohort than that calculated using the OPENSAFELY data. These risks were comparable to that found for an independent East London cohort (ETHICAL [13]) and is likely due to the high prevalence of these ethnicities in London compared to the national population and the increased risk of infection these groups face due to societal, cultural, and occupational pressures [31–33].

Ethnicity-specific data was not available for the place of Covid-19 associated death. As such, applying degrees of misspecification to ${\widehat{\varvec{\lambda }}}_{\varvec{b}}$ (parameter describing the effect of interaction between ethnicity and death on hospitalisation) was based on conjecture. News articles suggested hesitancy towards hospital care by some ethnic groups in the first wave of the pandemic [34]. Therefore ${\widehat{\varvec{\lambda }}}_{\varvec{b}}$ was adjusted to reduce the probability of hospitalisation in those from minority ethnicities who died.

The differences in log risk/RRs between the OPENSAFELY analysis and GSTT specific data (Table 2) provided guides for the level of misspecification which should be applied to each fitted parameter in Eq. 1. Comparison between the OPENSAFELY analysis and data for the Greater London area (Appendix Table 2) often suggested more extreme differences than that suggested when comparing the OPENSAFELY analysis to GSTT specific data (usually in the same direction). As a result, three levels of misspecification were applied to each parameter (Table 3) allowing for differing degrees of misspecification. These were: approximation of the log risk/RRs calculated in Table 2 (called 100%), a milder degree of misspecification (called 50%), and a more extreme degree of misspecification akin to that seen for the Greater London area demonstrated in Appendix Table 2 (called 200%). Each set of probabilities obtained were checked for appropriate expected changes which remained within the range of {0–1} prior to use. This resulted in some adjustments to the approximate values described here.

**Table 3:** The chosen values of misspecification applied to the GLM described in Eqs. 1 and 2 modelling the risk of Covid-19 associated hospitalisation based on ethnic group and the primary outcome death during hospitalisation (Eq. 1) or based on ethnic group, death during hospitalisation and Covid-19 wave as a covariate (Eq. 2). Values relating to 50%, 100% and 200% of predicted misspecification were applied to these estimates as part of the sensitivity analysis. ^a^misspecification was applied alongside a 100% misspecification of ${\widehat{\gamma }}_{1}$ to allow the probability of hospitalisation in those who died to be increased

**% Misspecification**	**Change in indicated parameter from Eqs. **1** & **2							
	${\widehat{\gamma }}_{0}$	${\widehat{\gamma }}_{1}$	${\widehat{\lambda }}_{a1}$	${\widehat{\lambda }}_{a2}$	${\widehat{\lambda }}_{a3}$	${\widehat{\lambda }}_{b1}$	${\widehat{\lambda }}_{b2}$	${\widehat{\lambda }}_{b3}$
50%	-0.05	-0.075	0.125^a^	0.125^a^	0.1^a^	-0.1	-0.1	-0.1
100%	-0.1	-0.15	0.25^a^	0.25^a^	0.2^a^	-0.2	-0.2	-0.2
200%	-0.2	-0.3	0.5^a^	0.5^a^	0.4^a^	-0.4	-0.4	-0.4
	**Change in indicated parameter from Eq. **2							
	${\widehat{\gamma }}_{2}$	${\widehat{\gamma }}_{3}$	${\widehat{\lambda }}_{c1}$	${\widehat{\lambda }}_{c2}$	${\widehat{\lambda }}_{c3}$	${\widehat{\lambda }}_{d1}$	${\widehat{\lambda }}_{d2}$	${\widehat{\lambda }}_{d3}$
50%	0.25^a^	-0.35	0.125^a^	-0.1^a^	0.125^a^	0.1^a^	0.1^a^	0.1^a^
100%	0.5^a^	-0.7	0.25^a^	-0.2^a^	0.25^a^	0.2^a^	0.2^a^	0.2^a^
200%	1.0^a^	-1.4	0.5^a^	-0.4^a^	0.5^a^	0.4^a^	0.4^a^	0.4^a^

The aim was to examine the misspecification of each parameter separately. However, adjustment of ${\widehat{\varvec{\lambda }}}_{\varvec{a}}$ would have resulted in the probability of hospitalisation in those who died to be above 1. To allow the suggested misspecification of ${\widehat{\varvec{\lambda }}}_{\varvec{a}}$ a 100% misspecification of ${\widehat{\gamma }}_{1}$ (value of -0.15) was also applied. This meant that the linear predictor (originally from Eq. 1) to which misspecification was applied became:

$${\eta }_{i}={\widehat{\gamma }}_{0}+({\widehat{\gamma }}_{1}-0.15){ death}_{\varvec{i}}+{\widehat{\varvec{\lambda }}}_{\varvec{a}}{\varvec{e}}_{\varvec{i}}+{{\widehat{\varvec{\lambda }}}_{\varvec{b}}{\varvec{e}}_{\varvec{i}}death}_{\varvec{i}}$$

After each level of misspecification was applied to the fitted model, the probability of hospitalisation for each ethnicity/survival status combination was recalculated, applied to the GSTT cohort and used as weights in the weighted analyses as per the original IPW analysis.

#### Part 3: Addition of covariates into the model equation

The methodology suggested by Thompson and Arah [25] allows the inclusion of covariates in the model determining probability of inclusion within a dataset. For a Covid-19 based analysis, one factor to consider is the wave of Covid-19. Research – including the OPENSAFELY analysis – indicates a time dependence for the risk of hospitalisation and mortality within different ethnic subgroups [11, 20, 27]. Therefore, accounting for this chance in risk when including data from multiple Covid-19 waves in a single IPW weighted analysis is vital.

As Mathur et al.’s analysis of the OPENSAFELY cohort includes separate data from the first (1st February to 3rd August 2020) and partial-second (1st September to 31st December 2020) wave of Covid-19 this same external data set can be used to develop IPWs from a model estimating the risk of hospitalisation based on patient ethnicity, survival status, and Covid-19 wave at admission. Adapting the initial model (Eq. 1), the risk can be calculated from a new GLM with the added elements indicated in blue (Eq. 2).


2

Following the same principles as described in the above sections, summarised data from the OPENSAFELY analysis across the first two waves of Covid-19 was applied to the model in Eq. 2 (Table 4). The calculated risks of hospitalisation for each ethnicity/wave/survival status group were inverted to create IPWs to apply to a weighted version of a Cox proportional hazards model with discharge as a competing risk. Degrees of misspecification likely to apply to the new model parameters in Eq. 2 were determined by comparing the OPENSAFELY data to other external sources (Appendix Tables 2 and 3). These model parameters were adjusted based on these comparisons (Table 3) and applied as part of a sensitivity analysis examining the effect of misspecification on the obtained results.

**Table 4:** The risk of hospitalisation with Covid-19 for each ethnic group in the first and second wave of Covid-19 as obtained from published analysis of the OPENSAFELY platform [20] and fitted parameters for the model in Eq. 2 that can be estimated from the OPENSAFELY data. OIM std error = standard errors derived from the observed information matrix variance estimators

**Ethnicity**		**Hypothesised risk of hospitalisation in wave 1**			**Hypothesised risk of hospitalisation in wave 2**		
		Survived	Died		Survived	Died	
White		0.00126	Assumed 0.90		0.00068	Assumed 0.90	
Black		0.00238	Assumed 0.90		0.00083	Assumed 0.90	
Asian		0.00197	Assumed 0.90		0.00140	Assumed 0.90	
Mixed/Other		0.00155	Assumed 0.90		0.00070	Assumed 0.90	
Unknown		0.00101	Assumed 0.90		0.00052	Assumed 0.90	
**Exponentiated fitted parameters ± OIM std. err.**							
${\widehat{\varvec{\gamma }}}_{0}$	0.0012 ± 0.0000	${\widehat{\gamma }}_{1}$	711.97 ± 6.659	${\widehat{\gamma }}_{2}$	0.5405 ± 0.0077	${\widehat{\gamma }}_{3}$	1.8501 ± 0.0286
${\widehat{\varvec{\lambda }}}_{\varvec{a}1}$	1.8806 ± 0.0679	${\widehat{\lambda }}_{b1}$	0.5317 ± 0.0221	${\widehat{\lambda }}_{c1}$	0.6446 ± 0.0448	${\widehat{\lambda }}_{d1}$	1.5513 ± 0.1276
${\widehat{\varvec{\lambda }}}_{\varvec{a}2}$	1.5615 ± 0.0356	${\widehat{\lambda }}_{b2}$	0.6404 ± 0.0167	${\widehat{\lambda }}_{c2}$	1.3144 ± 0.0467	${\widehat{\lambda }}_{d2}$	0.7608 ± 0.0309
${\widehat{\varvec{\lambda }}}_{\varvec{a}3}$	1.2241 ± 0.0510	${\widehat{\lambda }}_{b3}$	0.8169 ± 0.0407	${\widehat{\lambda }}_{c3}$	0.8321 ± 0.0610	${\widehat{\lambda }}_{d3}$	1.2018 ± 0.1086
${\widehat{\varvec{\lambda }}}_{\varvec{a}4}$	0.8017 ± 0.0136	${\widehat{\lambda }}_{b4}$	1.2474 ± 0.0231	${\widehat{\lambda }}_{c4}$	0.9492 ± 0.0278	${\widehat{\lambda }}_{d4}$	1.0535 ± 0.0332

## Results

### Part 1: Estimating the probability of Covid-19 associated hospitalisation in the first wave from national data

**Fig. 2:**
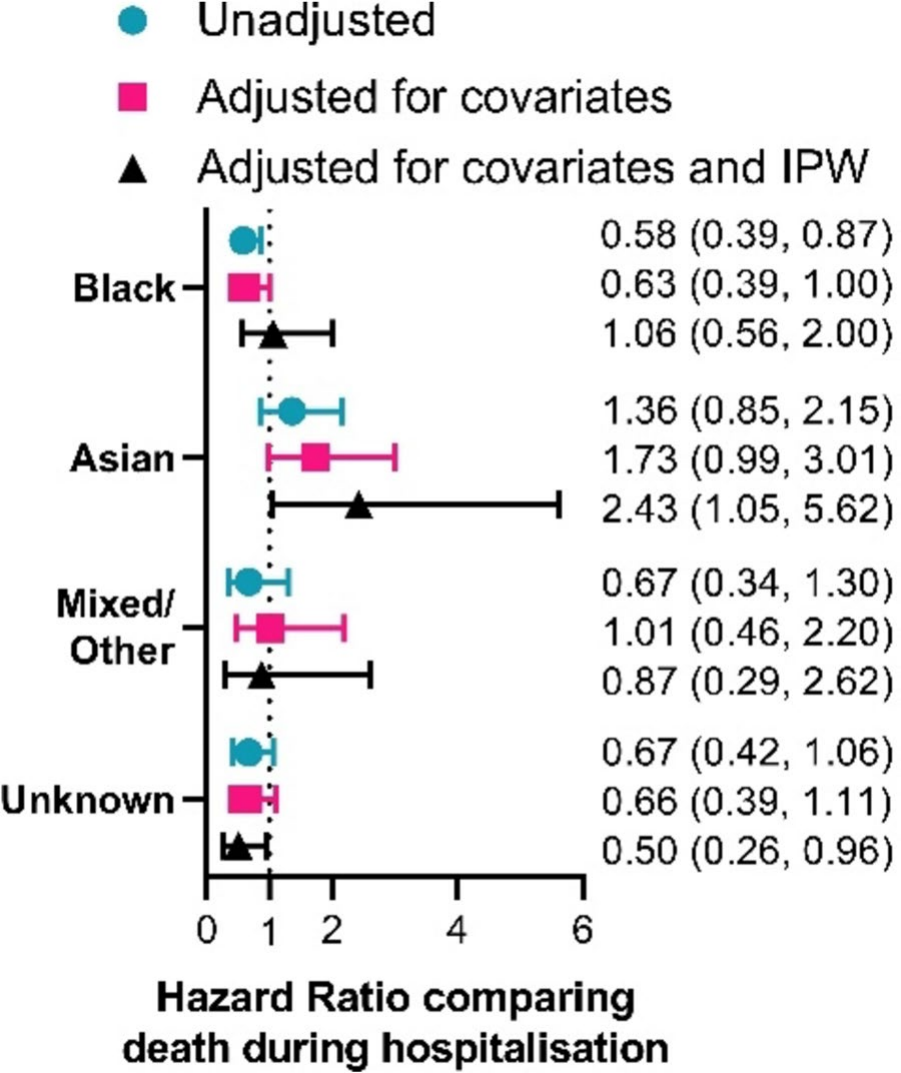
Summarised estimates for the effect of ethnic group (in comparison to White) on death during hospitalisation during the first wave of Covid-19. Estimates are obtained from an unadjusted analysis not accounting for covariates (green circles), an adjusted analysis containing 9 covariates (red squares), and a weighted analysis containing the 9 covariates and IPW based on calculated probabilities of hospitalisation (black triangles). The 9 covariates accounted for are: age, sex, IMD quintile, the presence of a DNR order, cardiovascular disease, COPD/emphysema, diabetes, chronic liver conditions and chronic kidney disease

Within a South-London cohort admitted to hospital during the first wave of Covid-19 (Fig. 2), examination of the relationship between ethnicity and Covid-19 associated mortality suggests a 42% (13%, 61%) reduction in risk of death in those of Black ethnicity (*p* = 0.008). This finding greatly differs from other studies examining a similar timeframe using less restricted cohorts suggesting likely bias within this analysis [17, 20, 26]. Accounting for covariates including medical history, age, sex and IMD does not account for the unexpected finding (hazard ratio (HR) = 0.63 (0.39, 1.00), *p* = 0.049).

Instead, use of IPW derived from external sources allowed the comparison of Black vs. White ethnicity to be revaluated resulting in a small increase in risk of death in those who were Black (HR = 1.06 (0.56, 2.00), *p* = 0.851). This hazard ratio now follows a similar trend to that seen in other studies [17, 20, 26] suggesting the use of IPW has corrected for potential sources of selection bias in this cohort.

Like the relationship between Black ethnicity and Covid-19 associated death, relationships of other ethnicities and survival were modified by the use of IPW to adjust for collider bias.

The effect of Asian ethnicity compared to White ethnicity on survival demonstrated some effect of confounding when comparing the unadjusted and adjusted models (green circle and red square, Fig. 2) with the estimated effects moving towards a more extreme hazard ratio after accounting for covariates (adjusted model: HR = 1.73 (0.99, 3.01), *p* = 0.052 vs. unadjusted model: HR = 1.36 (0.85, 2.15), *p* = 0.197). The use of IPW to account for selection biases, however, strengthens the estimated effect of Asian ethnicity (vs. White) on survival even further (HR = 2.43 (1.05–5.62), *p* = 0.038) allowing the findings to match the existing literature more closely [13, 17, 20, 26, 27].

The effect of Mixed/Other and Unknown ethnicities demonstrated less extreme changes in hazard ratios after adjusting for covariates followed by IPW to correct for collider bias (Fig. 2). However, this also matches existing studies which focus on an effect of Black and Asian ethnicity on Covid-19 outcomes in the first UK wave of the pandemic [12, 17, 20, 26].

### Part 2: Sensitivity analysis examining the effect of changes in estimated probabilities to match the local population

**Fig. 3:**
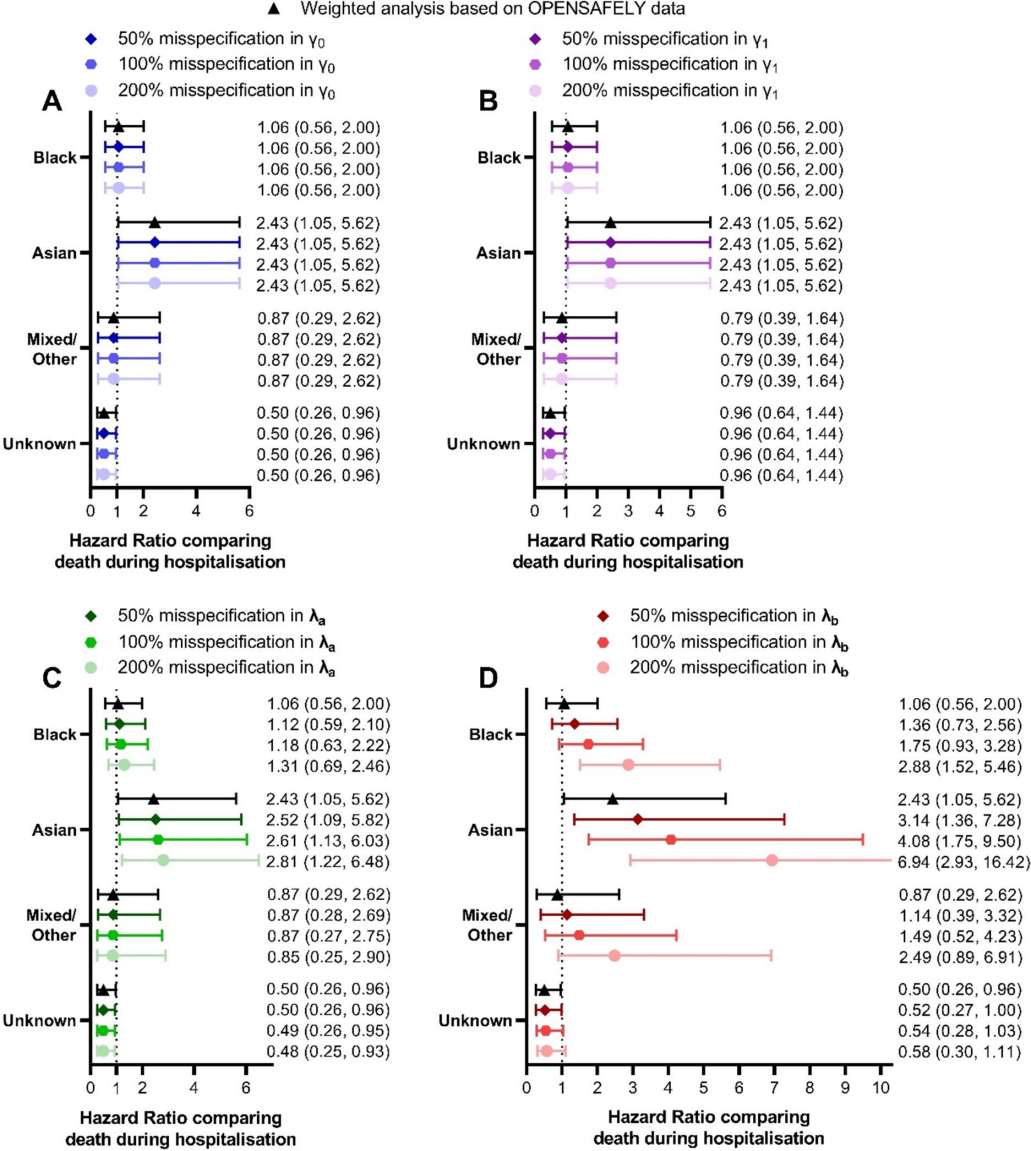
Changes seen to the estimated effect of ethnicity on death (recorded during hospitalisation) when completing sensitivity analysis to account for misspecification in parameters of the GLM modelling risk of Covid-19 associated hospitalisation based on ethnic group and survival status. Each parameter was adjusted based on three levels of misspecification: 50% (mild), 100% (expected) and 200% (extreme)

Applying these adjusted IPWs as part of the weighted survival analysis to examine the effect of misspecification in the relationship between Covid-19 associated hospitalisation and ethnicity/Covid-19 associated mortality found that adjustments to ${\widehat{\gamma }}_{0}$ (Fig. 3A), ${\widehat{\gamma }}_{1}$ (Fig. 3B), and ${\widehat{\varvec{\lambda }}}_{\varvec{a}}$ (Fig. 3C) had minimal effect on the estimates obtained from the analysis using IPW demonstrated in Fig. 2.

Reducing the probability of hospitalisation in those who died specifically in Black, Asian and Mixed/Other ethnicities (${\widehat{\varvec{\lambda }}}_{\varvec{b}}$) had a more dramatic effect on the estimates for the effect of each ethnic group on mortality (Fig. 3D). The estimated hazard ratios for all three ethnicities increased and there was more uncertainty about these estimates. The extent of the heightened hazard and the increased uncertainty was systematically enhanced as the degree of misspecification increased from 50 to 200%. Adjustments made to ${\widehat{\varvec{\lambda }}}_{\varvec{b}}$ had little effect on the estimated effect of Unknown ethnicity on death (Fig. 3D) – as expected because the probability of hospitalisation in those who died in this group was not adjusted.

Interestingly, the adjustment for misspecification in ${\widehat{\varvec{\lambda }}}_{\varvec{b}}$ allowed the estimated effect of Black ethnicity on mortality to reflect the situation reported in other studies [17, 20, 27] and the media during wave 1 of Covid-19 –an increased risk of death compared to those who are White. This differs from the results for the cohort from before applying IPWs to account for selection bias (Fig. 2) and highlights that in hospitalised cohorts, such as this one, there is a clear risk of inducing bias in the form of collider bias and other selection biases.

### Part 3: Addition of covariates into the model equation

**Fig. 4:**
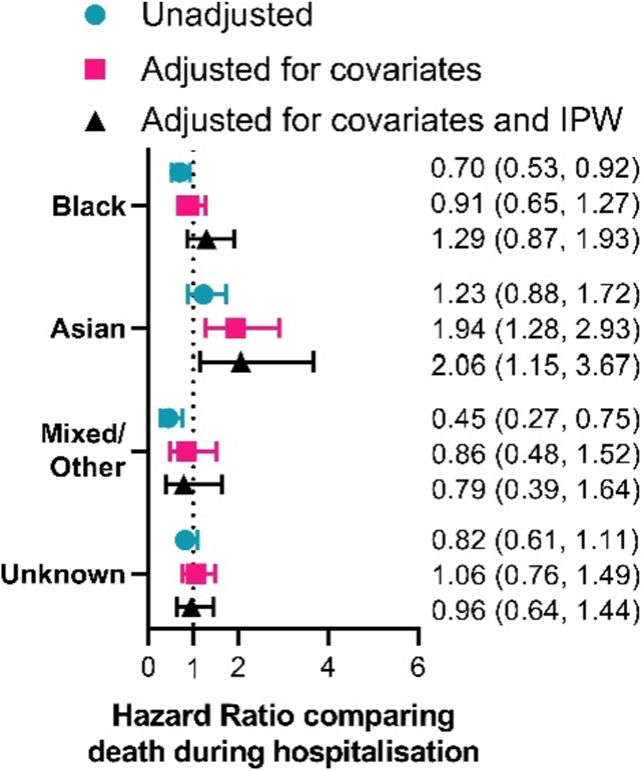
Summarised estimates for the effect of ethnic group (in comparison to White) on death during hospitalisation across the first two waves of Covid-19. Estimates are obtained from an unadjusted analysis not accounting for covariates (green circles), an adjusted analysis containing 9 covariates (red squares), and a weighted analysis containing the 9 covariates and IPW based on calculated probabilities of hospitalisation in each wave of Covid-19 (black triangles). The 9 covariates accounted for are: age, sex, IMD quintile, the presence of a DNR order, cardiovascular disease, COPD/emphysema, diabetes, chronic liver conditions and chronic kidney disease

The addition of data from the second wave of Covid-19 altered the results obtained from the initial analysis (Fig. 2) demonstrating how IPW in addition to adjusting for covariates improves estimation. All estimates now have more precision reflecting the larger cohort analysed (Fig. 4). Most point estimates have shifted to indicate a smaller/neutral effect of ethnicity on mortality with some exceptions. All point estimates fell within the 95% confidence intervals estimated from the wave 1 only cohort (Fig. 2).

This analysis continued to suggest a reduced risk of death in those of Black ethnicity (compared to White) in the unadjusted analysis (HR = 0.70 (0.53, 0.92), *p* = 0.012). This was corrected by adjustment for covariates (HR = 0.91 (0.65, 1.27), *p* = 0.575) and tended towards a small increase in mortality (HR = 1.29 (0.87, 1.93), *p* = 0.211) after the use of IPW to account for selection bias.

Asian ethnicity had a clear increased risk of mortality after adjustment for covariates (HR = 1.94 (1.28, 2.93), *p* = 0.002). Use of IPW did little to alter this finding demonstrating continued increased risk of mortality in those of Asian ethnicity (HR = 2.06 (1.15, 3.67), *p* = 0.014) as shown by other studies [13, 20, 27].

Inclusion of wave 2 in this analysis suggested a reduced risk of mortality in those with Mixed/Other ethnicity that was less evident in the initial analysis (HR = 0.45 (0.27, 0.75), *p* = 0.002). This reduction in risk was attenuated by adjustment for covariates and using IPW to correct for collider bias (HR = 0.79 (0.39, 1.64), *p* = 0.533). Meanwhile inclusion of wave 2 in this analysis suggested a lack of difference in mortality between those with Unknown ethnicity and those with known White ethnicity (unadjusted: HR = 0.82 (0.61, 1.11), *p* = 0.198; adjusted for covariates and IPW: HR = 0.96 (0.64, 1.44), *p* = 0.838) matching results seen in other studies [20].

#### Sensitivity analysis of the extended analysis

The sensitivity analysis examining misspecification in the model parameters for the GLM describing probability of hospitalisation while including wave as a covariate (Eq. 2) showed a similar pattern as the sensitivity analysis of the initial model.

**Fig. 5:**
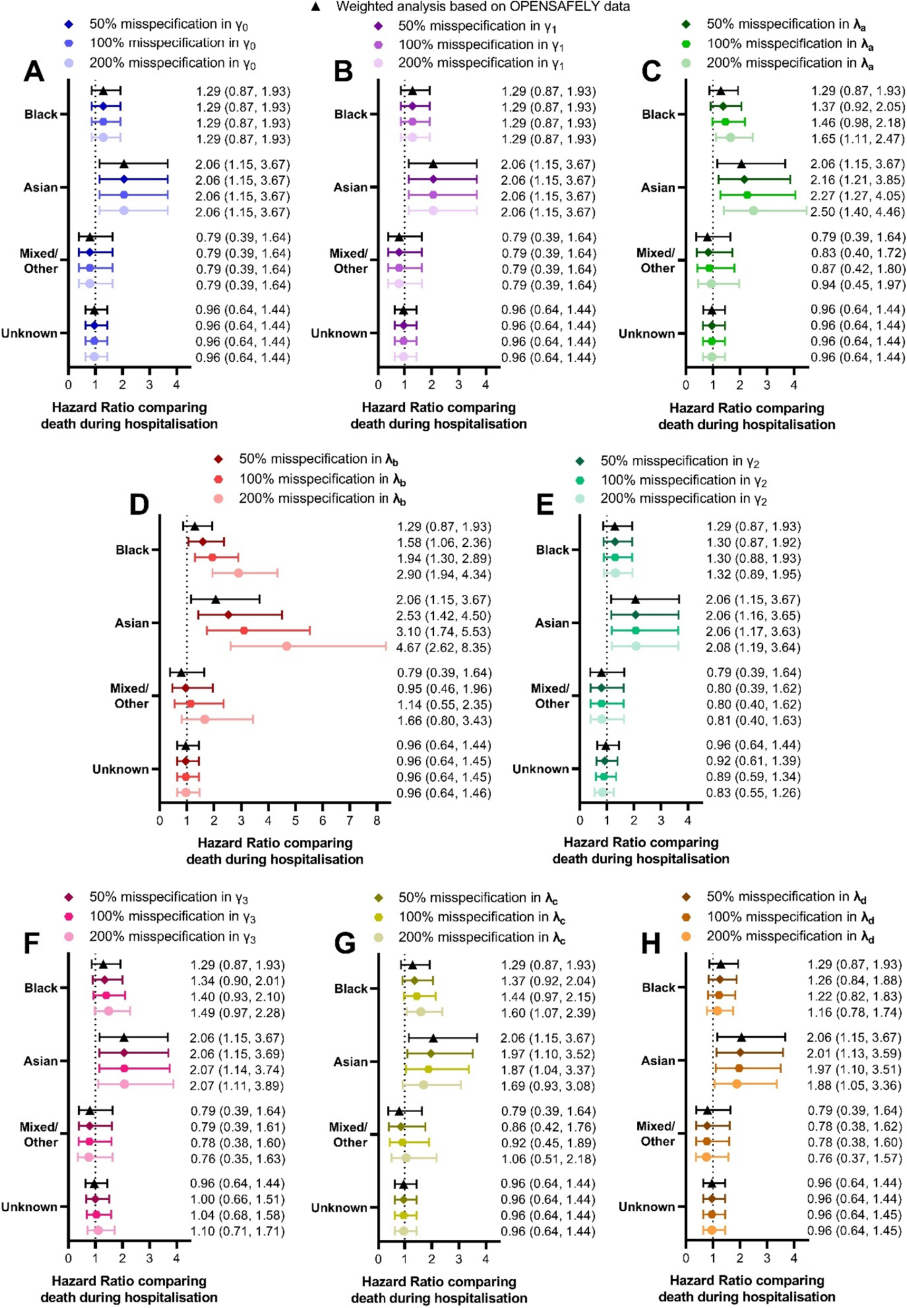
Changes seen to the estimated effect of ethnicity on death (recorded during hospitalisation) when completing sensitivity analysis to account for misspecification in parameters of the GLM modelling risk of Covid-19 associated hospitalisation based on ethnic group, Covid-19 wave and survival status. Each parameter was adjusted based on three levels of misspecification: 50% (mild), 100% (expected) and 200% (extreme)

Adjustments to ${\widehat{\gamma }}_{0}$ (Fig. 5A), ${\widehat{\gamma }}_{1}$ (Fig. 5B), ${\widehat{\gamma }}_{2}$ (Fig. 5E), and ${\widehat{\gamma }}_{3}$ (Fig. 5F) had minimal effect on the obtained estimates for the effect of ethnicity on mortality. Adjustments to ${\widehat{\varvec{\lambda }}}_{\varvec{a}}$ (Fig. 5C), ${\widehat{\varvec{\lambda }}}_{\varvec{c}}$ (Fig. 5G), and ${\widehat{\varvec{\lambda }}}_{\varvec{d}}$ (Fig. 5H) had small but notable effects on these estimates. Accounting for a large (200%) misspecification of ${\widehat{\varvec{\lambda }}}_{\varvec{a}}$ (related to the probability of hospitalisation in surviving individuals of minority ethnicities) was sufficient to increase the estimated risk of mortality in Black individuals compared to White, while adjustments to ${\widehat{\varvec{\lambda }}}_{\varvec{c}}$ and ${\widehat{\varvec{\lambda }}}_{\varvec{d}}$ (which related to ethnicity-specific effects on the risk of hospitalisation in wave 2) caused a reduction in the estimated hazard ratio comparing Asian and White ethnicity.

As before the only model parameter which had a substantial effect on the obtained results once misspecification was applied is ${\widehat{\varvec{\lambda }}}_{\varvec{b}}$ (Fig. 5D) which matches the pattern described above (Fig. 3D). Namely reducing the probability of hospitalisation in those who died specifically in Black, Asian and Mixed/Other ethnicities resulted in increases in the estimated hazard ratios for the relationship between mortality and these ethnic groups (compared to White ethnicity).

## Discussion

This study demonstrates that externally derived data can be used to develop IPW in order to create a weighted statistical analysis accounting for selection bias which is typical of studies during the Covid-19 pandemic. Within the example cohort, non-weighted analyses typically provided results that did not match findings from other UK-based studies [12, 13, 17, 20, 26]. The fact that this is a hospitalised cohort could be the key to this, creating a study prone to collider bias (Fig. 1). Weighted analysis using IPW instead allowed the results obtained from this cohort to be more representative of a non-hospitalised cohort as well as more comparable to the existing literature.

A key consideration in creating the IPWs used in this analysis was the choice of external data to use when modelling the probability of hospitalisation. The published analysis by Mathur et al. [20] of the OPENSAFELY platform data was deemed an appropriate choice as this assessed the risk of hospitalisation and death in an English community-based population of adults (18+) at risk of hospitalisation with Covid-19 in 2020 – a similar population and time period that individuals in this analysis were selected from. But the OPENSAFELY platform is a national cohort, while this is a London-based one with unique characteristics, including increased ethnic diversity [22] and an increased risk of Covid-19 infection [35]. This made the inclusion of a sensitivity analysis accounting for misspecification in the modelled relationship between ethnicity/wave/death and hospitalisation vital. Misspecification applied as part of this analysis was determined through comparisons of the OPENSAFELY data and other data sources. Most comparisons did not differentiate between ethnic groups or survival status due to lack of stratum specific data meaning that a certain degree of conjecture was required. To account for possible error in the comparisons and speculations made, multiple levels of misspecification were applied to each parameter. The sensitivities analyses demonstrated that large degrees of misspecification (200% of that predicted) would be required before the conclusions made differed from that described. In fact, misspecification in several model parameters demonstrated very little effect on the consistency of the obtained results, even when an initial model parameter (${\widehat{\gamma }}_{1}$) was based on an assumption later proved to be incorrect.

Only sequential levels of misspecification were chosen because it was found that broadly the rankings between ethnic groups for the probability of hospitalisation were consistent between the OPENSAFELY data and other data sources (Table 2, Appendix Tables 2 and 3). For the one exception to this finding (Appendix Table 3: in wave 2 those of Asian ethnicity local to GSTT were less likely to be hospitalised than those of Black ethnicity), the misspecification applied to ${\widehat{\varvec{\lambda }}}_{\varvec{c}}$ purposely reversed the rankings to fit the local data. Notably this change in rankings did not has a substantial effect on the obtained results (Fig. 5G). As a result, we are confident that the model based on OPENSAFELY platform data is likely to be appropriate for developing IPWs for this dataset.

One limitation of the misspecification methodology applied here is that misspecification was applied to each parameter independently. This was to determine if certain parameters were more susceptible to misspecification than other parameters as was found to be the case with only misspecification of ${\widehat{\varvec{\lambda }}}_{\varvec{b}}$ (probability of hospitalisation in those who died from minority ethnicities) have a substantial impact on the obtained results. An additional benefit is that it allowed for more control over how the probability of hospitalisation changed such that the probabilities could be kept within the required range of {0–1}. The exact method for creating externally derived IPWs should be examined in further studies. Firstly, this analysis was unable to incorporate additional covariates such as age or sex into the model determining the probability of hospitalisation which can be seen as a limitation. This was due to external datasets only being available as summary statistics without sufficient cross-tabulation. Simpler and so more obtainable methods for deriving IPWs exist based on the demographic differences between a sample and the target population which could account for such covariates. However, these have achieved mixed results [36]. Additionally, stabilisation methods [37] may be beneficial to analyses such as this one. The results of this analysis accepted large confidence intervals as a consequence of the large weights applied (due to low probabilities of hospitalisation) and the use of robust standard errors to maintain the type 1 error rate. These issues may be corrected by weight stabilisation [38].

Another factor that may have contributed to the large confidence intervals obtained is the categories of ethnicity used. Due to the small sample size, ethnicity was defined based on the broad categories employed by ONS. However, the Asian ethnic group, in particular, is a diverse population made up of multiple subgroups who have been shown to be impacted differently by Covid-19 [20, 27]. Notably South Asians make up 52.2% (140/268) of this cohort and this population have been demonstrated to have increased susceptibility to Covid-19.Despite the desire for additional considerations in the choice and methodology when applying weights, the need to use techniques such as these cannot be overstated in fields such as Covid-19. The relationship between Covid-19 severity and hospitalisation means that hospitalised cohorts can naturally only represent the most severe cases of Covid-19 [15] limiting the inferences can be drawn from these patient populations. Adding other selection pressures such as the provided example of increased Covid-19 infection and hospitalisation in minority ethnicities means that these cohorts are not representative of the wider UK population. Limiting selection biases such as collider bias [2, 6] and where possible correcting for these biases should be a vital part in Covid-19 methodology to ensure valid inferences are made and consistency within the published literature.

## Conclusion

In conclusion, this analysis demonstrates the use of inverse probability weighting derived from an external dataset to correct for collider bias present within cohorts of patients hospitalised with Covid-19. This correction allows an example data set exploring the relationship between ethnicity and Covid-19 associated mortality in a South London hospitalised cohort to be more consistent with the published literature.

### Supplementary Information

**Additional file 1.**

## Data Availability

The datasets generated and/or analysed during the current study are not publicly available due to the closure of the relevant databases and cloud-based services in line with the agreed ethical approvals. Further summary data is avaliable from the corresponding author on reasonable request.
