# Supplementary material for: Application of information from external data to correct for collider bias in a Covid-19 hospitalised cohort
Source: BMC Med Res Methodol. 2024 Jul 16;24:149. doi: 10.1186/s12874-023-02129-7 (PMC11250979; doi:10.1186/s12874-023-02129-7)
Supplement: Supplementary file 1 — Additional file 1. [file 12874_2023_2129_MOESM1_ESM.docx]

**Application of information from external data to correct for collider bias in a Covid-19 hospitalised cohort**

Annastazia E Learoyd^1^, Jennifer Nicholas^2^, Nicholas Hart^3,4^ and Abdel Douiri^1^

^1^ School of Life Course and Population Sciences, King College London, London, UK

^2^ Department of Medical Statistics, London School of Hygiene and Tropical Medicine, London, UK

^3^ Lane Fox Clinical Respiratory Physiology Research Centre, Guy's & St Thomas' NHS Foundation Trust, London, UK

^4^ Centre for Human and Applied Physiological Sciences, King's College London, UK


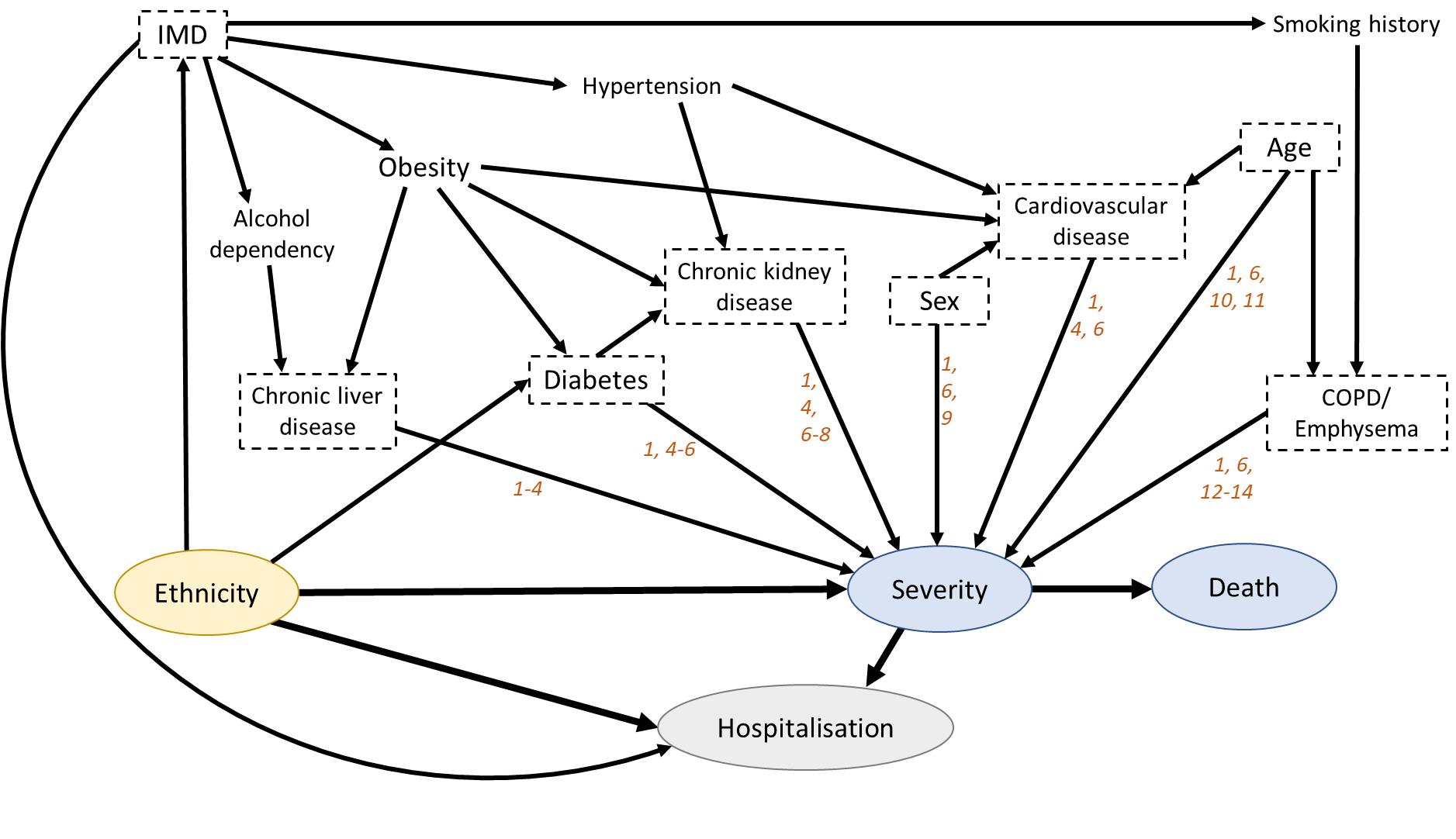


***Appendix Figure 1:*** Directed acyclic graph demonstrating the relationships between ethnicity (key exposure, yellow), Covid-19 severity/death (outcomes, blue), hospitalisation due to Covid-19 (collider, grey), and other relevant comorbidities. Chronic liver disease^1–4^, diabetes^1,4–6^, chronic kidney disease^1,4,6–8^, sex^1,6,9^, cardiovascular disease^1,4,6^, age^1,6,10,11^, and respiratory disease in the form of COPD and emphysema^1,6,12–14^ were corrected for in adjusted analyses along with index of multiple deprivation^1,15^ (indicated by dashed borders). These confounders were chosen based on evidence of an independent relationship with Covid-19 severity. IMD=Index of Multiple Deprivation. COPD=Chronic Obstructive Pulmonary Disease. Also published in Learoyd et al^16^.

**References informing DAG:**

1. Williamson, E. J. *et al.* Factors associated with COVID-19-related death using OpenSAFELY. *Nature* **584**, 430–436 (2020).

2. Mahamid, M. *et al.* Nonalcoholic fatty liver disease is associated with COVID-19 severity independently of metabolic syndrome: a retrospective case-control study. *Eur. J. Gastroenterol. Hepatol.* **33**, 1578–1581 (2021).

3. Singh, A., Hussain, S. & Antony, B. Non-alcoholic fatty liver disease and clinical outcomes in patients with COVID-19: A comprehensive systematic review and meta-analysis. *Diabetes Metab. Syndr.* **15**, 813 (2021).

4. Harrison, S. L., Buckley, B. J. R., Rivera-Caravaca, J. M., Zhang, J. & Lip, G. Y. H. Cardiovascular risk factors, cardiovascular disease, and COVID-19: an umbrella review of systematic reviews. *Eur. Hear. J. - Qual. Care Clin. Outcomes* **7**, 330–339 (2021).

5. Kaminska, H. *et al.* Impact of diabetes mellitus on in-hospital mortality in adult patients with COVID-19: a systematic review and meta-analysis. *Acta Diabetol.* **58**, 1101–1110 (2021).

6. Biswas, M., Rahaman, S., Biswas, T. K., Haque, Z. & Ibrahim, B. Association of Sex, Age, and Comorbidities with Mortality in COVID-19 Patients: A Systematic Review and Meta-Analysis. *Intervirology* **64**, 36–47 (2021).

7. Dashtban, M. *et al.* Predicting and Validating Risk of Pre-Pandemic and Excess Mortality in Individuals With Chronic Kidney Disease. *SSRN Electron. J.* (2021) doi:10.2139/SSRN.3970707.

8. Wang, B. *et al.* The Involvement of Chronic Kidney Disease and Acute Kidney Injury in Disease Severity and Mortality in Patients with COVID-19: A Meta-Analysis. *Kidney Blood Press. Res.* **46**, 17–30 (2021).

9. Nguyen, N. T. *et al.* Male gender is a predictor of higher mortality in hospitalized adults with COVID-19. *PLoS One* **16**, 1–6 (2021).

10. Singhal, S., Kumar, P., Singh, S., Saha, S. & Dev, A. B. D. Clinical features and outcomes of COVID-19 in older adults: a systematic review. *BMC Geriatics* **21**, 321 (2021).

11. Starke, K. R. *et al.* The isolated effect of age on the risk of COVID-19 severe outcomes: a systematic review with meta-analysis. *BMJ Glob. Heal.* **6**, e006434 (2021).

12. Gerayeli, F. V. *et al.* COPD and the risk of poor outcomes in COVID-19: A systematic review and meta-analysis. *EClinicalMedicine* **33**, 100789 (2021).

13. Pardhan, S., Wood, S., Vaughan, M. & Trott, M. The Risk of COVID-19 Related Hospitalsation, Intensive Care Unit Admission and Mortality in People With Underlying Asthma or COPD: A Systematic Review and Meta-Analysis. *Front. Med.* **8**, 668808 (2021).

14. Reyes, F. M. *et al.* Assessment of the Association of COPD and Asthma with In-Hospital Mortality in Patients with COVID-19. A Systematic Review, Meta-Analysis, and Meta-Regression Analysis. *J. Clin. Med.* **10**, 2087 (2021).

15. Elliott, J. *et al.* COVID-19 mortality in the UK Biobank cohort: revisiting and evaluating risk factors. *Eur. J. Epidemiol.* **36**, 299–309 (2021).

16. Learoyd, A. E., Nicholas, J., Hart, N. & Douiri, A. Revisiting ethnic discrepancies in a COVID-19 hospitalised cohort. *J Clin Epidemiol* **4356**, 00161–0 (2023).

## Extra methodology

The statistical analysis described here was primarily completed in the secure King’s Health Partners Rosalind high-performance computer infrastructure running Jupyter Notebook 6.0.3, R 3.6.3 and Python 3.7.6.

Algorithm for performing analysis

*Section 1: Producing IPWs from external data*

1. Using external data sources produce a table with the following components:

| Ethnicity | Survival_status | Wave | Num_risk | Num_hosp |
| --- | --- | --- | --- | --- |
| White | 0 [=Survived] | 1 | 13741 | 10870464 |
| White | 1 [=Died] | 1 | 6763 | 7514 |
| Black | 0 [=Survived] | 1 | 810 | 340644 |

1. Import table into statistical software.
2. Perform a GLM for grouped binary data with the following variables:
   - Link function: log
   - Family: binomial
   - Outcome: Num_hosp
   - Predictors: Ethnicity, Survival_status, Wave
   - Number at risk (i.e. in each group): Num_risk
3. In order to perform the sensitivity analysis keep a record of the estimated values for all parameters.
4. Determine the probability of hospitalisation for each strata either using the GLM model or directly from the data (both methods should produce the same values).
5. Produce a variable for the IPWs as 1/probability calculated in step 5.

*Section 2: Producing adjusted IPWs for sensitivity analysis*

1. Write an equation based on Equation 1 using the estimates calculated in step 4.
2. Adjust the first parameter in Equation 1 ($\hat{\gamma}_{0}$) by the required degree specified for the sensitivity analysis (i.e. add the value for $\hat{\gamma}_{0}$ in Table 3 to the estimate obtained in step 4). Produce a variable describing the new probabilities of hospitalisation for each group.
3. Repeat step 8 for each parameter.
4. If using multiple levels of misspecification repeat steps 8 and 9 for each level of misspecification.

End result should be a dataset with a variable for the original probabilities of hospitalisation obtained from the external dataset and a variable for each extra analysis performed as part of the sensitivity analysis. [Named the probability dataset].

*Step 3: Analysis of the patient cohort*

1. Import cleaned dataset for patient cohort into statistical software.
2. Merge the patient dataset and the probability dataset.
3. Perform analysis of outcome (time to in-hospital death) without IPWs. Details:
   - Model type: Competing risks with robust standard errors
   - Outcome: Binary indicator of survival status
   - Competing risk: Indictor of discharge status
   - Time variable: Time until death or discharge or end of follow-up
   - Predictors: Ethnicity (key exposure), Age centred on average (here mean=59.7), Sex, IMD quintile, indicator of cardiovascular disease, indicator of diabetes, indicator of kidney disease, indicator of liver disease, indicator of COPD/emphysema, time dependent indicator of application of DNR order
4. Repeat step 13 (analysis of outcome) with the addition of sampling weights. Use variable indicating probability of hospitalisation as the sampling weight.
5. Repeat step 14 with each variable generated for the sensitivity analysis.

| Variable/Interaction | OPENSAFELY | | | London | | | Difference in ln(RR) | Informs |
| --- | --- | --- | --- | --- | --- | --- | --- | --- |
|  | Risk | | RR | Risk | | RR |  |  |
| Wave |  | | *(NHS reported cases/ONS estimated population)* | | | | |  |
| 1 (Feb/March – Aug 2020) | 0.00187 | |  | 0.00248 | |  | **ln(Risk)=0.28** | $\hat{\gamma}_{0}$ |
| 2 (Sept – Dec 2020/May 2021) | 0.00107 | | 0.57 | 0.00522 | | 2.10 | **1.30** | $\hat{\gamma}_{2}$ |
| Wave 1: Outcome | No Death: *(NHS reported discharges/ONS estimated population)*  Death: *(ONS death occurrences involving COVID-19)* | | | | | | |  |
| No Death | 0.00121 | |  | 0.00180 | |  |  |  |
| Death | Assumed 0.90 | | 744 | 0.73476 | | 408 | **-0.60** | $\hat{\gamma}_{1}$ |
| Wave 2: Outcome |  | |  |  | |  |  |  |
| No Death | 0.00066 | |  | 0.00406 | |  |  |  |
| Death | Assumed 0.90 | | 1,363 | 0.81174 | | 200 | **-1.92** |  |
|  |  | | | **Wave 2 – Wave 1** | | | **-1.32** | $\hat{\gamma}_{3}$ |
| Wave 1: Ethnicity |  | | *(ETHICAL cohort/ONS estimated population)* | | | | |  |
| White | 0.00185 | |  | 0.00092 | |  |  |  |
| Black | 0.00307 | | 1.66 | 0.00257 | | 2.79 | **0.52** | $\hat{\lambda}_{a1}$ |
| Asian | 0.00257 | | 1.39 | 0.00155 | | 1.68 | **0.19** | $\hat{\lambda}_{a2}$ |
| Mixed/Other | 0.00188 | | 1.02 | 0.00137 | | 1.50 | **0.39** | $\hat{\lambda}_{a3}$ |
| Wave 2: Ethnicity |  | |  |  | | |  |  |
| White | 0.00107 | |  | 0.00210 | |  |  |  |
| Black | 0.00102 | | 0.95 | 0.00452 | | 2.15 | **0.82** |  |
| Asian | 0.00182 | | 1.70 | 0.00596 | | 2.84 | **0.51** |  |
| Mixed/Other | 0.00082 | | 0.76 | 0.00375 | | 1.79 | **0.86** |  |
|  |  |  | | **Wave 2 – Wave 1** | Black | | **0.30** | $\hat{\lambda}_{c1}$ |
|  |  |  | |  | Asian | | **0.32** | $\hat{\lambda}_{c2}$ |
|  |  |  | |  | Mixed/Other | | **0.47** | $\hat{\lambda}_{c3}$ |

**Appendix Table 1:** Risk of Covid-19 associated hospitalisation during two Covid-19 waves for those with different outcomes/ethnicities as calculated for the OPENSAFELY platform cohort^20^ and the Greater London area. Data used to calculate the risks for the Greater London area are indicated in italics. The ETHICAL cohort^13^ relates to a population in Newham, Tower Hamlets, Waltham Forest - ONS estimates for this region was applied to calculate risks. Risk Ratios (RR) compare risks to applicable reference groups: Wave 1, No Death or White ethnicity. Differences in ln(Risk) or ln(RR) were used as an additional reference of the level of misspecification to apply to each indicated parameter (Informs column) during the sensitivity analysis. Comparable data to inform misspecification of ${\hat{\boldsymbol{\lambda}}}_{\boldsymbol{a}}$ and ${\hat{\boldsymbol{\lambda}}}_{\boldsymbol{d}}$ was not available.

| Variable/Interaction | OPENSAFELY | | | GSTT | | | Difference in ln(RR) | Informs |
| --- | --- | --- | --- | --- | --- | --- | --- | --- |
|  | Risk | | RR | Risk | | RR |  |  |
| Wave |  | | *(NHS reported cases/ONS estimated population)* | | | | |  |
| 1 (Feb/March – Aug 2020) | 0.00187 | |  | 0.00177 | |  | **ln(Risk)=-0.05** | $\hat{\gamma}_{0}$ |
| 2 (Sept – Dec 2020/May 2021) | 0.00107 | | 0.57 | 0.00137 | | 0.77 | **0.30** | $\hat{\gamma}_{2}$ |
| Wave 1: Outcome | No Death: *(NHS reported discharges/ONS estimated population)*  Death: *(ONS death occurrences involving COVID-19)* | | | | | | |  |
| No Death | 0.00121 | |  | 0.00104 | |  |  |  |
| Death | Assumed 0.90 | | 744 | 0.76359 | | 734 | **-0.01** | $\hat{\gamma}_{1}$ |
| Wave 2: Outcome |  | |  |  | |  |  |  |
| No Death | 0.00066 | |  | 0.00116 | |  |  |  |
| Death | Assumed 0.90 | | 1,363 | 0.78941 | | 681 | **-0.70** |  |
|  |  | | | **Wave 2 – Wave 1** | | | **-0.69** | $\hat{\gamma}_{3}$ |
| Wave 1: Ethnicity |  | | *(Analysed cohort/ONS estimated population)* | | | | |  |
| White | 0.00185 | |  | 0.00047 | |  |  |  |
| Black | 0.00307 | | 1.66 | 0.00118 | | 2.48 | **0.40** | $\hat{\lambda}_{a1}$ |
| Asian | 0.00257 | | 1.39 | 0.00089 | | 1.87 | **0.30** | $\hat{\lambda}_{a2}$ |
| Mixed/Other | 0.00188 | | 1.02 | 0.00054 | | 1.14 | **0.11** | $\hat{\lambda}_{a3}$ |
| Wave 2: Ethnicity |  | |  |  | | |  |  |
| White | 0.00107 | |  | 0.00101 | |  |  |  |
| Black | 0.00102 | | 0.95 | 0.00222 | | 2.20 | **0.84** |  |
| Asian | 0.00182 | | 1.70 | 0.00183 | | 1.82 | **0.07** |  |
| Mixed/Other | 0.00082 | | 0.76 | 0.00133 | | 1.33 | **0.55** |  |
|  |  |  | | **Wave 2 – Wave 1** | Black | | **0.44** | $\hat{\lambda}_{c1}$ |
|  |  |  | |  | Asian | | **-0.23** | $\hat{\lambda}_{c2}$ |
|  |  |  | |  | Mixed/Other | | **0.44** | $\hat{\lambda}_{c3}$ |

**Appendix Table 2:** Risk of Covid-19 associated hospitalisation during two Covid-19 waves for those with different outcomes/ethnicities as calculated for the OPENSAFELY cohort^20^ and the GSTT catchment area. Data informing the risk for the GSTT catchment area indicated in italics. Risk Ratios (RR) compare risks to applicable reference groups: Wave 1, No Death or White ethnicity. Differences in ln(Risk) or ln(RR) were used as an additional reference of the level of misspecification to apply to each indicated parameter (Informs column) during the sensitivity analysis. Comparable data to inform misspecification of ${\hat{\boldsymbol{\lambda}}}_{\boldsymbol{a}}$ and ${\hat{\boldsymbol{\lambda}}}_{\boldsymbol{d}}$ was not available.

## Cohort characteristics

Of the 3,133 patients admitted to hospital, 53.4% were male and the mean age was 59.71±18.80 (Appendix Table 3). Overall, 63.8% (n=2,000) of patients had a medical history of at least one comorbidity of interest, with the most common being cardiovascular conditions, followed by diabetes and chronic kidney disease. Representation of ethnic groups were as follows: 40.4% White, 24.9% Black, 8.6% Asian, 8.6% Mixed/Other and 17.7% Unknown. Differences were seen between ethnic groups in the majority of characteristics, including age, sex, and presence of comorbidities (Appendix Table 3). There was a trend towards a difference in ethnic distributions across wave one and wave two of Covid-19 ($\chi_{4}^{2}$=9.00, p=0.061) with the first wave containing a higher proportion of Black people and the second wave containing more people from the Mixed/Other and Unknown ethnic groups.

The median hospital stay length was 5.76 days (ranging from 0.01 to 243.61 days). 89.6% of patients (n=2,806) stayed in hospital for 30 days or less. During this time 304 (9.7%) patients died, and 513 (16.4%) patients were admitted to ICU with a median ICU stay of 8.2 days (range: 0.0 to 136.2 days). An additional 52 (1.7%) patients died after this 30 day period meaning that overall 356 (11.4%) patients died.

Deaths occurred steadily over time from hospital admission until ~20 days with the mortality rate declining thereafter (Appendix Figure 2A). Survival curves of each ethnic group (Appendix Figure 2B) suggest a decreased risk of death in Black and Mixed/Other ethnic groups and a comparable risk of death in Asians and Whites. These survival curves match the unadjusted hazard ratios provided in the main text (Figure 4).

| Patient Characteristics | All patients | Ethnicity groups | | | | | Comparison | |
| --- | --- | --- | --- | --- | --- | --- | --- | --- |
|  |  | White | Black | Asian | Mixed/Other | Unknown | χ^2^ | p-value |
| Total num. (%) of patients | 3,133 | 1,265 (40.4%) | 779 (24.9%) | 268 (8.6%) | 268 (8.6%) | 553 (17.7%) |  |  |
| Num. (%) patients - Wave 1 | 1,010 | 415 (41.1%) | 274 (27.1%) | 87 (8.6%) | 72 (7.1%) | 162 (16.0%) |  |  |
| Num. (%) patients - Wave 2 | 2,123 | 850 (40.0%) | 505 (23.8%) | 181 (8.5%) | 196 (9.2%) | 391 (18.4%) |  |  |
| *Covariates* | | | | | | | | |
| *Index of Multiple Deprivation* | |  |  |  |  |  |  |  |
| Rank [Median (IQR)] | 9,324 (6591-13,839) | 9,929 (7,088-14,996) | 8,105 (6,008-11,635) | 8,161 (6,422-12,982) | 8,894 (6,731-12,694) | 9,939 (6,731-15,898) | 69.05 | **<0.001** |
| Quintile (%) |  |  |  |  |  |  | 92.15 | **<0.001** |
| 1 (Lowest) | 775 (24.7%) | 267 (21.1%) | 236 (30.3%) | 75 (28.0%) | 64 (23.9%) | 133 (24.1%) |  |  |
| 2 | 1,528 (48.8%) | 617 (48.8%) | 402 (51.6%) | 128 (47.8%) | 141 (52.6%) | 240 (43.4%) |  |  |
| 3 | 498 (15.9%) | 221 (17.5%) | 111 (14.2%) | 40 (14.9%) | 38 (14.2%) | 88 (15.9%) |  |  |
| 4 | 227 (7.2%) | 100 (7.9%) | 27 (3.5%) | 19 (7.1%) | 19 (7.1%) | 62 (11.2%) |  |  |
| 5 (Highest) | 105 (3.4%) | 60 (4.7%) | 3 (0.4%) | 6 (2.2%) | 6 (2.2%) | 30 (5.4%) |  |  |
| Age (Mean±SD) | 59.71±18.80 | 64.06±18.81 | 57.56±18.33 | 56.13±17.71 | 53.38±17.98 | 57.58±18.29 | 127.83 | **<0.001** |
| Male Sex (%) | 1,674 (53.4%) | 691 (54.6%) | 369 (47.4%) | 153 (57.1%) | 134 (50.0%) | 327 (59.1%) | 22.16 | **<0.001** |
| *DNARCPR applied* |  |  |  |  |  |  |  |  |
| Number (%) | 693 (22.1%) | 348 (27.5%) | 139 (17.8%) | 56 (20.9%) | 32 (11.9%) | 118 (21.3%) | 46.15 | **<0.001** |
| Time to DNARCPR (days) | 1.3 (0.2-9.8) | 1.1 (0.2-10.3) | 1.2 (0.3-8.5) | 1.8 (0.2-15.2) | 0.8 (0.2-5.3) | 2.8 (0.3-11.0) | 4.30 | 0.367 |
| Cardiovascular conds (%) | 1,710 (54.6%) | 776 (61.3%) | 454 (58.3%) | 136 (50.7%) | 123 (45.9%) | 221 (40.0%) | 85.04 | **<0.001** |
| COPD/Emphysema (%) | 262 (8.4%) | 176 (13.9%) | 27 (3.5%) | 9 (3.4%) | 18 (6.7%) | 32 (5.8%) | 89.72 | **<0.001** |
| Diabetes (%) | 875 (27.9%) | 326 (25.8%) | 276 (35.4%) | 96 (35.8%) | 56 (20.9%) | 121 (21.9%) | 49.63 | **<0.001** |
| Kidney conditions (%) | 631 (20.1%) | 263 (20.8%) | 210 (27.0%) | 53 (19.8%) | 32 (11.9%) | 73 (13.2%) | 50.63 | **<0.001** |
| Liver conditions (%) | 82 (2.6%) | 42 (3.3%) | 20 (2.6%) | 9 (3.4%) | 4 (1.5%) | 7 (1.3%) | 8.33 | 0.080 |
| *Death during hospital stay* | |  |  |  |  |  |  |  |
| Number (%) | 356 (11.4%) | 166 (13.1%) | 72 (9.2%) | 42 (15.7%) | 16 (6.0%) | 60 (10.8%) | 20.19 | **<0.001** |
| Time to death (days) | 11.1 (5.7-21.8) | 11.8 (5.9-22.2) | 10.8 (5.3-18.9) | 11.0 (5.5-23.2) | 8.2 (3.1-10.9) | 11.8 (7.4-23.1) | 5.44 | 0.245 |
| Time to censor (days) | 5.0 (1.4-12.7) | 6.1 (1.9-14.9) | 4.8 (1.4-12.6) | 3.9 (1.1-8.7) | 3.3 (0.8-10.1) | 4.7 (1.2-11.9) |  |  |

***Appendix Table 3:*** Patient characteristics. Times reported as Median (IQR). Censored means patient discharged without experiencing event. IQR=Interquartile Range. DNARCPR= “do not attempt resuscitation” order. COPD=chronic obstructive pulmonary disease.


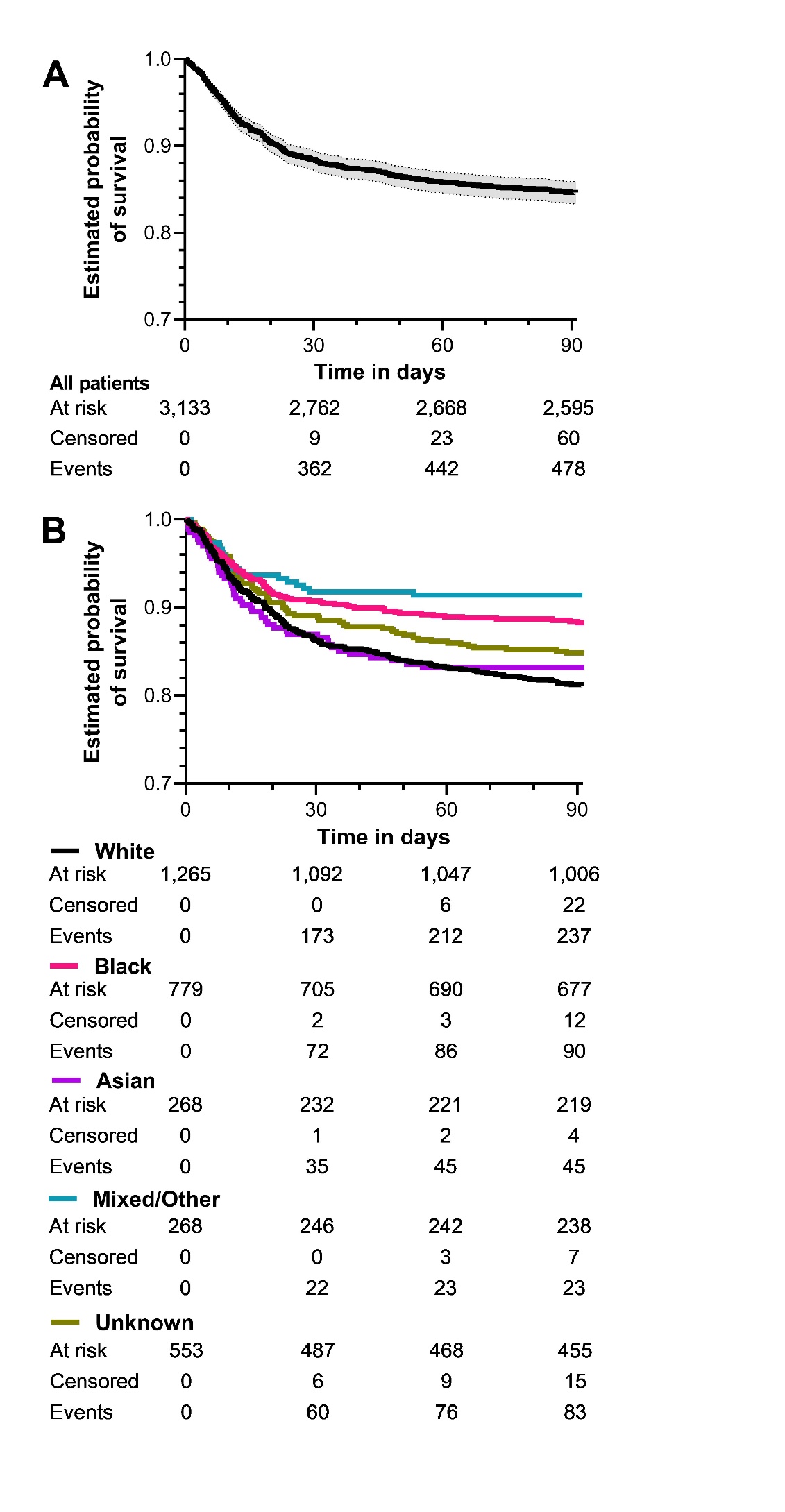
***Appendix Figure 2:*** Kaplan Meier plots with associated risk tables for the probability of death over days from hospital admission (in all patients (A) and the following ethnic groups (B): White (black), Black (red), Asian (purple), Mixed/Other (turquoise) and Unknown (brown).
